# Differences in technology innovation R&D performance creation behavior between for-profit institutions and not-for-profit institutions

**DOI:** 10.1186/s40064-016-2092-x

**Published:** 2016-04-14

**Authors:** Sungmin Park

**Affiliations:** Department of Business Administration, Baekseok University, Cheonan, 330-704 Republic of Korea

**Keywords:** Data splitting, For-profit institutions, Not-for-profit institutions, R&D collaboration, Stepwise performance creation, Successive binary logistic regression

## Abstract

The present study compares the performance creation behavior between for-profit institutions and not-for-profit institutions within a national technology innovation research and development (R&D) program. Based on the stepwise performance creation chain structure of typical R&D logic models, a series of successive binary logistic regression models is newly proposed. Using the models, a sample of *n* = 2076 completed government-sponsored R&D projects was analyzed. For each institution type, its distinctive behavior is diagnosed, and relevant implications are suggested for improving the R&D performance.

## Introduction

One of the major research topics of research and development (R&D) performance evaluation is the discussion of determining factors on R&D performance. Some literature attempts to measure their own influence quantitatively. In particular, a number of preceding literature focus on the following subjects: (1) the role of government subsidy for creating R&D performance associated with government-sponsored R&D projects (i.e., GSPs) (Jaffe 1996; David et al. 2000; Hall 2002), (2) the relationship between the firm size and R&D performance (Scherer and Ross 1990; Graves and Langowits 1993; Rothwell and Dodgson 1994), and (3) R&D performance improvement through R&D collaboration (Kogut 1988; Das and Teng 2000; Hagedoorn et al. 2000; Van Aken and Weggeman 2000; Reagans and Zuckerman 2001; Caloghirou and Hondroyiannis 2003; Narula and Duysters 2004; Nieto and Santamaria 2007; Hillman et al. 2009).

However, a more detailed classification of determining factors on R&D performance, and relevant procedures for the performance comparisons according to the classified factors, are very limited so far. Research is needed to verify differences in R&D performance according to the following classifications (Wernerfelt 1984; Barney 2002; Grimaldi and Tunzelmann 2003): (1) by institution types (i.e., types of R&D lead agencies) such as universities, research laboratories and companies, (2) by R&D objectives such as basics, application and commercialization, (3) by the sizes of tangible R&D inputs such as R&D budget, period and workforce, and (4) by the R&D-intensive industry areas such as biotechnology, electronics, chemicals, etc. Recently, some literature has mentioned specific topics regarding the extent to which R&D inputs exert substantial influence on R&D performance, the identification of the relationship between R&D inputs and key performance factors, the verification of R&D performance differences among types of institutions and R&D collaboration, and so forth (Shipp et al. 2005; Ruegg 2006; Åström et al. 2010; KEIT 2010, 2011, 2013; Elg and Håkansson 2012).

Regarding the national technology innovation R&D programs referred below, empirical studies have addressed whether statistically significant differences exist in R&D performance between institution types and between R&D collaboration types. In the empirical analyses of related studies, a common research limitation was the incomplete panel samples that could not fully consider the time lag between R&D inputs and the performance (Wu et al. 2006; Guan and Chen 2010; Chen et al. 2011). In addition, the inherent scarcity of GSPs achieving R&D performance might be another reason why researchers have not collected proper datasets.

The present study conducts an empirical analysis aiming to verify differences in R&D performance behavior between for-profit institutions and not-for-profit institutions within a national technology innovation R&D program. Based on the analyses of the present study, some policy implications are derived to help practitioners for accomplishing their own R&D management objectives effectively. A sample of *n* = 2076 completed GSPs during the recent five performance follow-up survey years (2008 to 2012) is analyzed. Data are collected from a representative national technology innovation R&D program, the Industry Technology Innovation Program (ITIP) administered by the Korean government’s Ministry of Trade, Industry and Energy (MOTIE). In particular, the whole sample is split into two mutually exclusive datasets to compare R&D performance behavior between the two types of institution more accurately. Also, the present study proposes a new analysis framework using successive binary logistic regression models, in which the inherent characteristics of observations (i.e., completed GSPs) can be reflected properly. This new methodology shows how to deal with the R&D performance creation success-failure binary characteristic. The present study is organized as follows. “Background and literature review” section states the background and literature review, “Research model” section explains the research model, and “Empirical analysis” section presents the empirical analyses. Finally, conclusions are summarized in “Conclusions” section. Additionally, in the “Appendix”, all the mathematical details are elaborated associated with the design of successive binary logistic regression models.

## Background and literature review

Generally, public sector R&D performance is evaluated based on a typical R&D logic model, and the efficiency, effectiveness, relevance and sustainability of GSPs are analyzed quantitatively using various methods. Consequently, the results and implications from the performance evaluation can be reflected in the decision-making process regarding R&D programs’ planning, deployment and budget allocations (Wholey 1983; Bickman 1987; Wholey 1987; McLaughlin and Jordan 1999; Ruegg and Feller 2003; WK Kellogg Foundation WKKF 2004). Lately, in addition to the quantitative efficiency perspective, the qualitative effectiveness viewpoint is underscored in the field of R&D performance evaluation with the consideration of a clear relationship between R&D inputs and crucial performance created by GSPs (Ruegg 2006; KISTEP 2011; MKE·KIAT 2012; STAR METRICS 2014). In national R&D programs’ planning and deployment stage, effective government subsidy allocations are demanded by reflecting the performance differences between institution types and between R&D collaboration types (KEIT 2010, 2011, 2013; OMB·OSTP 2012; OSTP 2012).

Regarding some national technology innovation R&D programs, typical R&D logic models were developed such as the Advanced Technology Program (ATP) logic model of the U.S. Department of Commerce (DOC) (Ruegg and Feller 2003) and the Research and Technology Development and Deployment Program (RTDDP) logic model of the U.S. Department of Energy (DOE) (McLaughlin and Jordan 1999). Representative national technology innovation R&D programs can be found such as the ATP under the DOC, the Industrial Technology Development Program (ITDP) administered by the Ministry of Economic Affairs (MEA) with the Taiwanese government, and the Knowledge Economy Technology Innovation Program (KETIP) conducted by the Ministry of Knowledge Economy (MKE) with the Korean government (Ruegg and Feller 2003; Shipp et al. 2005; Ruegg 2006; Hsu and Hsueh 2009; KEIT 2010, 2011, 2013).

David et al. (2000) investigated the role of government subsidies in the private R&D investments by scrutinizing a total of 33 previous studies from 1966 to 2000. The majority of the papers examined argued for a net complementary effect in which a government subsidy facilitated private R&D investment (17 papers). The remainder described a net expulsive effect in which the government subsidy only replaced the private R&D investment (11 papers). Up to date, a clear agreement has not been reached yet on the relationship between government subsidy and the amount of private R&D investment (Jaffe 1996; Hall 2002).

As one of the critical influencing factors regarding performance enhancement, R&D collaboration between researchers or between research groups may be considered. It is known that the utilization of diverse perspectives through R&D collaboration contributes to the improvement of R&D performance (Van Aken and Weggeman 2000; Reagans and Zuckerman 2001; Caloghirou and Hondroyiannis 2003). Furthermore, R&D collaboration has the advantage of providing an environment where each research entity can combine complementary resources of R&D collaboration participants in terms of allowing access to information and knowledge held within each entity. In general, universities and research laboratories have comparative capabilities for basic and applied research, and for-profit companies tend to focus more on commercialization-oriented R&D projects. In this context, it is recognized that R&D performance can be enhanced readily through R&D collaboration by multiple competent institutions (Kogut 1988; Das and Teng 2000; Hagedoorn et al. 2000; Narula and Duysters 2004; Nieto and Santamaria 2007; Hillman et al. 2009). Specifically, many preceding studies reported that key measures representing the level of technology innovation of the companies were greatly improved by carrying out external R&D collaboration (Fritsch and Lukas 2001; Belderbos et al. 2004; Laursen and Salter 2006; Ortega-Argilés et al. 2009; Chen et al. 2011; Gronum et al. 2012; Berchicci 2013; Esteve-Pérez and Rodríguez 2013; Robin and Schubert 2013).

The Office of Management and Budget (OMB) and the Office of Science and Technology Policy (OSTP) under the U.S. Government emphasized the need for R&D collaboration associated with the 2014 federal R&D budget compilation and execution procedure (OMB·OSTP 2012). In addition, the ATP accepted applications from single companies and joint ventures. For-profit companies could apply as single applicants to receive an award up to $2 million USD over 3 years to cover project costs (Ruegg and Feller 2003). Also, among the initial ATP’s 50 completed GSPs, 42 GSPs (84 %) were conducted through R&D collaboration.

Most of the literature used nonnormal statistical models. Because of the inherent scarcity of GSPs achieving R&D performance, and the extreme skew to the right distribution, the literature seems to adopt non-normal techniques such as Tobit regression models to cope with the censored data characteristics, binary logistic regression models to deal with the performance creation success-failure binary characteristic, and so on (Fritsch and Lukas 2001; Laursen and Salter 2006; Berchicci 2013; Robin and Schubert 2013). Related to measuring the relative efficiency and the total productivity changes in R&D programs, some prior studies provided excellent classifications on R&D inputs and the performance factors to be considered in the performance evaluation (Meng et al. 2006; Wu et al. 2006; Sharma and Thomas 2008; Hsu and Hsueh 2009; Guan and Chen 2010; Chen et al. 2011; Park 2014).

Additionally, some research papers were reported associated with the relationship between R&D management and technology innovation. Tan et al. (2015) presented a comparative impact analysis on collaborative research in Malaysia using journal articles published in the 10-year period spanning, the years 2000–2009. Bacchiocchi and Montobbio (2009) estimated the process of diffusion and decay of knowledge from university, public laboratories and corporate patents. Hu (2009) investigated the extent to which East Asia had become a source of international knowledge diffusion and whether such diffusion was localized to the region. Branstetter and Ogura (2005) emphasized on the use of the knowledge generated by university-based scientists. Meantime, related to measuring efficiency of R&D programs, Chen et al. (2004) assessed the R&D efficiency of 31 computer-related companies in Taiwan. They examined the total efficiency, technical efficiency and scale efficiency respectively, and revealed the correlations between inputs and outputs. Osawa and Murakami (2002), Eilat et al. (2008), Hashimoto and Haneda (2008), and Cullmann et al. (2012) analyzed the number of patents as one of the output variables for evaluating R&D performance. Also, Kim et al. (2009) argued that the number of patents per R&D expenditure declined with the firm size (i.e., the firm sales) for both pharmaceutical and semiconductor companies. Lamperti et al. (2015) examined the impact of science parks on growth and innovativeness of affiliated firms. They found that both patenting activity and R&D investments were actively sustained by the presence and quantity of research centers within the park.

## Research model

**Fig. 1:**
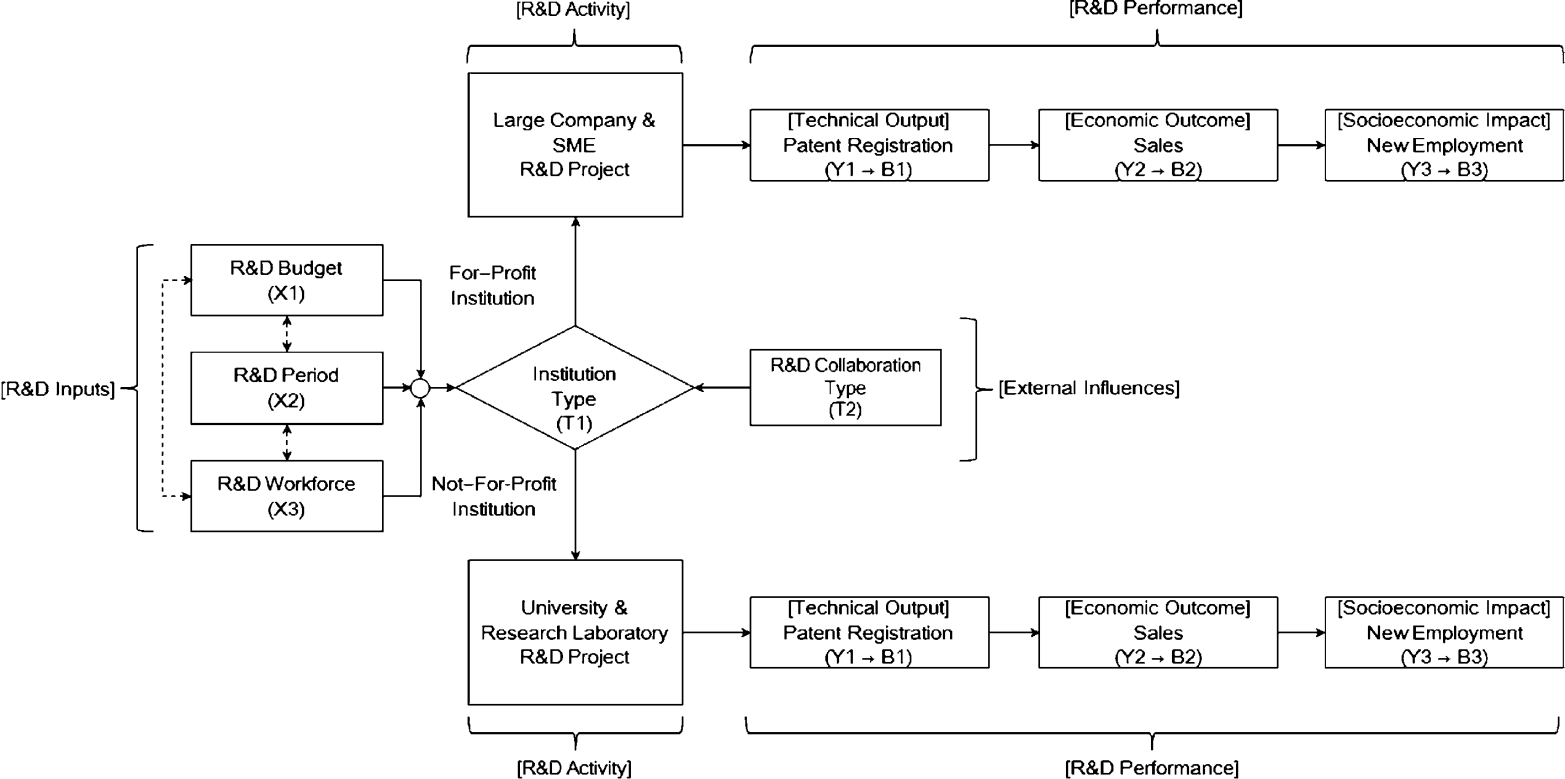
A research model with R&D inputs, performance and external influences

Figure 1 shows the research model of the present study in which important measures of R&D inputs and performance are organized based on the related literature (Tong and Frame 1994; Werner and Souder 1997; McLaughlin and Jordan 1999; Ruegg and Feller 2003; WK Kellogg Foundation WKKF 2004; Meng et al. 2006; Wu et al. 2006; Bitman and Sharif 2008; Sharma and Thomas 2008; Hsu and Hsueh 2009; Guan and Chen 2010; Chen et al. 2011; Park 2014). In Fig. 1, drawn with squares, multiple R&D inputs and performance factors comprises the stepwise chain structure of the research model. Ruegg and Feller (2003) and Hsu and Hsueh (2009) presented representative R&D inputs and performance factors for a GSP-level performance analysis. Some external influence factors were pointed out such as institution types, R&D collaboration, the internal R&D capability and the accumulated knowledge and experience of institutions (Geuna et al. 2003; Stephan 2010). It was reported that intangible R&D inputs (i.e., accumulated research experience, educational and training efforts for human resources by institutions, etc.) demonstrated a positive influence on R&D performance enhancement (Bowman 1992; Parikh 2001; Lee et al. 2005). As described in “Empirical analysis” section, the research model in Fig. 1 is designed as a parsimonious model composed of quantitative characteristics of GSPs analyzed in the present study. In particular, based on the literature such as Ruegg and Feller (2003), Shipp et al. (2005), Ruegg (2006), Wu et al. (2006), Hsu and Hsueh (2009), Guan and Chen (2010), Chen et al. (2011), the key performance factors suggested include published articles, patent applications and registrations, patents used, profited commercialization sales, new employment, and so forth. Also, we can find discussions on the typical R&D performance creation pattern conformed to the stepwise chain structure from the literature.

The total of eight variables describe the overall characteristics of each observation (i.e., GSP), which included R&D inputs, performance and external influence factors as shown in Fig. 1. For the input variables, three characteristics are considered: R&D Budget (X1), R&D Period (X2) and R&D Workforce (X3). The three performance variables analyzed are Patent Registration (Y1), Sales (Y2) and New Employment (Y3). The present study considers two additional external influence variables, Institution Type (T1) and R&D Collaboration Type (T2). The three performance variables (Y1, Y2 and Y3) are converted into three corresponding binary variables, B1, B2 and B3 respectively, to deal with the sample characteristics as explained in “Empirical analysis” section. For example, for the *i*th observation, if the condition of Y1_*i*_ > 0 is satisfied (i.e., the case of patent registration performance creation success), then the corresponding patent registration performance creation success-failure binary variable B1_*i*_ is defined as 1 (i.e., B1_*i*_ = 1 if Y1_*i*_ > 0 and B1_*i*_ = 0 otherwise). Meanwhile, the variables are selected based on the representative factors examined in the aforementioned literature closely related to GSP-level performance evaluation, and the data availability; the reliability of the sample is fully verified beforehand.

Two external influence variables T1 and T2 are defined as follows. First of all, T1 is a 4-level categorical variable. According to the institution type of the *i*th observation, T1 has four different values: (1) T1_*i*_ = L (Large Company), (2) T1_*i*_ = U (University), (3) T1_*i*_ = R (Research Laboratory), and (4) T1_*i*_ = S (Small and Medium-Sized Enterprise, SME). T2 is a 3-level categorical variable. T2 is classified into three separate values based on the R&D collaboration type of the *i*th observation: (1) T2_*i*_ = Sg (Single Institution R&D), (2) T2_*i*_ = Cs (R&D Collaboration with the Same Type Institution), and (3) T2_*i*_ = Cd (R&D Collaboration with the Different Type Institution). Specifically, X1 is the pure amount of government R&D subsidy, and the institution type, denoted by T1_*i*_ = R, refers to government-funded research laboratory only. Furthermore, the four institution types are categorized into two broader institution types: for-profit institution (i.e., large company and SME) and not-for-profit institution (i.e., university and research laboratory). If only the *i*th observation reports R&D collaboration between the two heterogeneous institution types, then T2_*i*_ is equal to Cd (i.e., R&D collaboration between for-profit institutions and not-for-profit institutions). On the other hand, T2_*i*_ = Cs means that the *i*th observation is conducted by R&D collaboration between the homogeneous institution types.

In particular, based on the assumption that R&D performance creation behavior can be different between for-profit institutions and not-for-profit institutions, the present study attempts to divide the whole sample of *n* = 2076 described in “Empirical analysis” section into two mutually exclusive subordinate datasets as follows: (1) the first dataset of *n*_*1*_ = 1637 with for-profit institutions, and (2) the second dataset of *n*_*2*_ = 439 with not-for-profit institutions. In addition to the whole sample, these two subordinate datasets are analyzed separately for more accurate comparisons of the performance behavior between the two types of institutions. Meanwhile, it is not desirable that all the mathematical details are embedded in the narrative at this point. Therefore, not to wade through all the detailed equations, the mathematical details are arranged in the appendix associated with the design of successive binary logistic regression models.

## Empirical analysis

### Description of the sample

As mentioned briefly above, the sample analyzed in the present study is a set of completed GSPs within a representative national technology innovation R&D program (i.e., ITIP) administered by the MOTIE with the Korean government over the recent five performance follow-up survey years (2008–2012). Initially, the sample consisted of 6267 completed GSPs. Even though the completion years of each GSP were slightly different, this sample can be regarded as fully considering the time lag between R&D inputs and the performance.

For the initial sample, the first data collection was carried out by investigating two national R&D databases provided by Korean government agencies such as the Project Management System (PMS) of the Korea Institute for Advancement of Technology (KIAT) and the eR&D of the Korea Evaluation Institute of Industrial Technology (KEIT). For the second data investigation stage, the data obtained from the two databases were verified, and missing data from the first data collection stage were gleaned using the National Science and Technology Information Service (NTIS) database administered by the Korea Institute of S&T Evaluation and Planning (KISTEP) (KISTI 2008; MKE 2008; MST·OSTI 2008). After the first two data collection processes, an offline survey was implemented to verify the data reliability associated with the sales as well as to obtain missing data of the R&D budget in particular. Consequently, the sample of *n* = 2067 completed GSPs (i.e., 33.13 % of the initial sample) was prepared.

Table 1 shows the descriptive statistics of the sample. As seen, the three continuous performance variables, Y1, Y2 and Y3, are severely skewed to the right. For example, Y2 has the largest coefficient of variation (CoefVar), and its CoefVar is equal to 4.71. With respect to the three continuous performance variables, the number of observations with a value greater than zero (i.e., the number of observations achieving performance) are as follows: (1) the short-term, technical output variable Y1, 905 (905/2076 × 100 = 43.59 %), (2) the mid-term, economic outcome variable Y2, 818 (39.40 %), and (3) the socioeconomic impact variable Y3, 560 (26.97 %). An interesting phenomenon was that the number of observations creating performance decreases monotonically when the position of each performance variable moves forward along with the research model’s chain path from the starting to the ending points. These proportions exactly coincide with the means of the three binary variables B1, B2 and B3 (Mean = 0.44, 0.39 and 0.27 respectively). The inherent scarcity of observations achieving performance can be confirmed because the medians of these three continuous performance variables are zero. In preparing the sample, the exchange rate of 1000 Won/$1 USD was applied to the raw data to convert monetary units.

**Table 1:** Descriptive statistics of the whole sample (*n* = 2076)

Variable	Name	Type	Unit and count (%)	Mean	SD	CoefVar	Median	Max	Skewness
R&D budget	X1	Continuous	(USD$ 10^6^)	2.00	2.40	1.20	1.25	27.00	2.90
R&D period	X2	Continuous	(Years)	3.16	1.73	0.55	3.00	9.84	1.00
R&D workforce	X3	Continuous	(Man-Years)	20.66	20.34	0.98	15.00	119.00	1.85
Patent registration	Y1	Continuous		2.00	4.47	2.24	0.00	39.00	4.04
Sales	Y2	Continuous	(USD$ 10^6^)	2.82	13.28	4.71	0.00	142.32	7.36
New employment	Y3	Continuous		3.41	11.60	3.40	0.00	115.00	6.03
Institution type	T1	Multinomial							
		L^a^	365 (17.58 %)						
		U^b^	151 (7.27 %)						
		R^c^	288 (13.87 %)						
		S^d^	1272 (61.27 %)						
		Total	2076 (100.00 %)						
R&D collaboration type	T2	Multinomial							
		Sg^e^	207 (9.97 %)						
		Cs^f^	566 (27.26 %)						
		Cd^g^	1303 (62.76 %)						
		Total	2076 (100.00 %)						
Binary conversion of Y1	B1	Binary		0.44					
		0	1171 (56.41 %)						
		1	905 (43.59 %)						
		Total	2076 (100.00 %)						
Binary conversion of Y2	B2	Binary		0.39					
		0	1258 (60.60 %)						
		1	818 (39.40 %)						
		Total	2076 (100.00 %)						
Binary conversion of Y3	B3	Binary		0.27					
		0	1516 (73.03 %)						
		1	560 (26.97 %)						
		Total	2076 (100.00 %)						

^a^ *L* large company

^b^ *U* University

^c^ *R* Research laboratory

^d^ *S* SME

^e^ *Sg* single institution R&D

^f^ *Cs* R&D collaboration with the same type institution

^g^ *Cd* R&D collaboration with the different type institution

Regarding the external influence variable T1, the sample composition proportions are as follows: (1) T1 = L, 365 (365/2076 × 100 = 17.58 %), (2) T1 = U, 151 (7.27 %), (3) T1 = R, 288 (13.87 %), and (4) T1 = S, 1272 (61.27 %). Hence, *n*_1_ = 1637 observations (78.85 %) were conducted by for-profit institutions (i.e., large companies and SMEs), and *n*_2_ = 439 observations (21.15 %) were carried out by not-for-profit institutions (i.e., universities and research laboratories). Thus, the sample composition proportions are rather asymmetric. Approximately, the sample composition proportions are divided as 80 versus 20 % between for-profit institutions and not-for-profit institutions.

The external influence variable T2 has the sample composition proportions as follows: (1) T2 = Sg, 207 (207/2076 × 100 = 9.97 %), (2) T2 = Cs, 566 (27.26 %), and (3) T2 = Cd, 1303 (62.76 %). It is noted that the majority of the sample is composed of observations adopting R&D collaboration with the different types of institutions. In addition, Table 1 shows descriptive statistics such as mean, standard deviation (SD), CoefVar, median, maximum value (Max) and skewness regarding the three R&D input variables X1, X2 and X3.

### R&D input variables’ correlation analysis

When a correlation exists among the R&D input variables X1, X2 and X3, the multicollinearity impairs the precision of the estimated regression coefficients as a whole. When a full model including all these input variables together is estimated, the standard errors of the estimated regression coefficients usually tend to be inflated drastically. Therefore, the stability of the estimated regression models cannot be ensured. In a general linear regression analysis, the degree of multicollinearity can be measured by Variance Inflation Factor (VIF). Because of the nonnormal characteristics in the present study, three kinds of correlation coefficients among the input variables are scrutinized: the parametric Pearson’s *r* and the nonparametric Kendall’s τ_*B*_ and Spearman’s ρ_s_ (Table 2). As seen in Table 2, strong correlations exist among these input variables, as expected. In particular, the largest correlation coefficients are found between X1 and the remaining two input variables, and all nine correlation coefficients in Table 2 have their own P values at 0.000***. The asterisk marks, *, **, *** indicate statistical significance at the significance level α = 10 %, 5 %, 1 % respectively. Hereafter, assuming that X1 is a representative input variable, a reduced model is analyzed associated with the input variable X1 only.

**Table 2:** Correlation coefficients of R&D input variables (the whole sample, *n* = 2076)

	X1 (R&D budget)	X2 (R&D period)
X2 (R&D period)		
Pearson’s *r*	0.408	
(P value)	(0.000***)	
Kendall’s τ_*B*_	0.510	
(P value)	(0.000***)	
Spearman’s ρ_s_	0.673	
(P value)	(0.000***)	
X3 (R&D workforce)		
Pearson’s *r*	0.578	0.084
(P value)	(0.000***)	(0.000***)
Kendall’s τ_*B*_	0.350	0.111
(P value)	(0.000***)	(0.000***)
Spearman’s ρ_s_	0.476	0.148
(P value)	(0.000***)	(0.000***)

*, **, *** Indicate statistical significance at the significance level α = 10, 5, 1 % respectively

### Logistic regression analysis: the whole sample (n = 2076)

#### Model structure

Table 3 shows the results from analyzing three successive binary logistic regression models from Model (1) to Model (3) using the whole sample of *n* = 2076 observations. For example, in Model (1), the case of the response variable B1 = 1 is defined as the reference case. As for the two external influence variables, the levels of T1 = L and T2 = Sg are defined as the reference levels. According to the research model’s performance chain structure, Model (1) is extended to Model (2) by adding a predictor variable B1 that is the response variable of the preceding model, Model (1). Consequently, in Model (3), the two additional predictor variables, B1 and B2, are included compared with Model (1). In Model (3), the levels of B1 = 0 and B2 = 0 are defined as the reference levels.

**Table 3:** Successive binary logistic regression analyses (the whole sample, *n* = 2076)

		Model (1)		Model (2)		Model (3)	
Response variable	(Level)	B1	(0, 1^a^)	B2	(0, 1^a^)	B3	(0, 1^a^)
Level	(Count)	0	(1171)	0	(1258)	0	(1516)
		1	(905)	1	(818)	1	(560)
		Total	(2076)	Total	(2076)	Total	(2076)
Predictor variable	(Level)	X1		X1		X1	
		T1	(L^b^, U, R, S)	T1	(L^b^, U, R, S)	T1	(L^b^, U, R, S)
		T2	(Sg^b^, Cs, Cd)	T2	(Sg^b^, Cs, Cd)	T2	(Sg^b^, Cs, Cd)
				B1	(0^b^, 1)	B1	(0^b^, 1)
						B2	(0^b^, 1)

Variable	Level	Coefficient (Z value)	Odds ratio	Coefficient (Z value)	Odds ratio	Coefficient (Z value)	Odds ratio
Intercept		−1.256 (−6.28)		−0.910 (−4.55)		−3.940 (−12.24)	
X1		0.283 (10.04***)	1.33	0.028 (1.15)	1.03	0.021 (0.57)	1.02
T1	U	0.343 (1.65*)	1.41	−1.770 (−5.48***)	0.17	−0.942 (−1.88*)	0.39
	R	0.225 (1.30)	1.25	−0.927 (−4.77***)	0.40	−0.307 (−1.06)	0.74
	S	0.187 (1.43)	1.21	0.702 (5.39***)	2.02	0.433 (2.29**)	1.54
T2	Cs	0.060 (0.33)	1.06	0.021 (0.12)	1.02	0.038 (0.15)	1.04
	Cd	0.419 (2.53**)	1.52	−0.014 (−0.08)	0.99	0.516 (2.19**)	1.68
B1	1			0.376 (3.79***)	1.46	0.420 (3.06***)	1.52
B2	1					3.603 (21.65***)	36.72
Model significance test							
Log Likelihood		−1322.902		−1286.288		−727.612	
Chi-Sq		197.967		211.403		965.432	
DF		6		7		8	
P value		0.000***		0.000***		0.000***	
Measures of association							
Somers’ D		0.37		0.34		0.76	
Goodman–Kruskal γ		0.37		0.34		0.77	
Kendall’s τ_*A*_		0.18		0.16		0.30	

Prediction pair type		Count	Proportion	Count	Proportion	Count	Proportion
Concordant pairs		720,302	0.680	674,699	0.656	743,362	0.876
Discordant pairs		332,881	0.314	329,685	0.320	96,802	0.114
Tied pairs		6572	0.006	24,660	0.024	8796	0.010
Total		1,059,755	1.000	1,029,044	1.000	848,960	1.000

^a^Reference case (i.e., Success)

^b^Reference level

#### Model diagnosis

In Table 3, to examine the significance of Model (1) accompanied with a total of six predictor variables, the likelihood ratio test was carried out. In contrast with Model (1), the null hypothesis model including only the intercept term can be estimated. In the likelihood ratio test, the deviance difference between Model (1) and the null hypothesis model is calculated as Chi-Sq ($\chi^{ 2}$) = ${\text{D}}_{{{\text{Fitted}}\; ( 1 )}}$–${\text{D}}_{\text{Null}}$  = 197.967 and *P* value = 0.000*** where ${\text{D}}_{{{\text{Fitted}}\; ( 1 )}}$ and ${\text{D}}_{\text{Null}}$ denote the deviance of Model (1) and the null hypothesis model respectively. Hence, the null hypothesis model can be rejected, and Model (1) is determined to be significant. In summary, all three models from Model (1) to Model (3) achieve the model significance based on the likelihood ratio test statistics.

Meanwhile, three measures of association of Model (3) are calculated to check the prediction capability: (1) Somer’s D = 0.76, (2) Goodman–Kruskal γ = 0.77, and (3) Kendall’s τ_A_ = 0.30. In practice, these measures of association can be referred to in the comparison with the remaining two models, Model (1) and Model (2). In Model (3), all three measures of association are greater than the corresponding values of the two other models, so the prediction capability of Model (3) is better than the two preceding models, Model (1) and Model (2).

Regarding Model (3), among the 848,960 (= 560 × 1516) pairs of (success, failure) observations, the number of concordant pairs (743,362; 87.6 %) is much larger than the number of discordant pairs (96,802; 11.4 %). In this binary logistic regression analysis, a concordant pair indicates that the pair with the predicted probability of the success observation is larger than the predicted probability of the failure observation. Inversely, the discordant pair is defined as the pair whose predicted probability of the failure observation is higher than that of the success observation. In terms of the concordant and discordant pair counts, Model (3) also gains good predictive power on the probability for B3. Among the three models, Model (3) has the largest proportion of concordant pairs (87.6 %), and Model (1) has the second largest proportion (68.0 %). The smallest proportion of concordant pairs (65.6 %) is found in Model (2). Therefore, the two models, Model (3) and Model (1), show better prediction capabilities, which also agrees with the interpretation of the measures of association.

As shown in Tables 4 and 5, all three models have model significance in terms of the likelihood ratio tests. Compared with the corresponding models in Table 3, the three models in Table 4 have reduced degrees of freedom (DF) such as 4, 5 and 6 respectively due to the reduction of the levels of the external influence variable T1. Additionally, in Model (3) of Table 5, the external influence variable T2 is converted into a 2-level categorical variable, so the model has DF = 5 (i.e., the total of five predictor variables). Consistently, based on the measures of association and prediction pair types’ proportions, the prediction capability of Model (3) is excellent in Tables 4 and 5.

**Table 4:** Successive binary logistic regression analyses (for-profit institutions, *n*_1_ = 1637)

		Model (1)		Model (2)		Model (3)	
Response variable	(Level)	B1	(0, 1^a^)	B2	(0, 1^a^)	B3	(0, 1^a^)
Level	(Count)	0	(966)	0	(884)	0	(1118)
		1	(671)	1	(753)	1	(519)
		Total	(1637)	Total	(1637)	Total	(1637)
Predictor variable	(Level)	X1		X1		X1	
		T1	(L^b^, S)	T1	(L^b^, S)	T1	(L^b^, S)
		T2	(Sg^b^, Cs, Cd)	T2	(Sg^b^, Cs, Cd)	T2	(Sg^b^, Cs, Cd)
				B1	(0^b^, 1)	B1	(0^b^, 1)
						B2	(0^b^, 1)

Variable	Level	Coefficient (Z value)	Odds ratio	Coefficient (Z value)	Odds ratio	Coefficient (Z value)	Odds ratio
Intercept		−1.063 (−4.71)		−0.873 (−3.98)		−3.977 (−11.28)	
X1		0.242 (6.44***)	1.27	−0.015 (−0.42)	0.99	−0.016 (−0.32)	0.98
T1	S	0.130 (0.96)	1.14	0.644 (4.76***)	1.90	0.372 (1.87*)	1.45
T2	Cs	−0.097 (−0.47)	0.91	0.078 (0.40)	1.08	0.060 (0.22)	1.06
	Cd	0.359 (1.84*)	1.43	−0.008 (−0.04)	0.99	0.512 (1.98**)	1.67
B1	1			0.514 (4.87***)	1.67	0.497 (3.40***)	1.64
B2	1					3.733 (19.79***)	41.79
Model significance test							
Log likelihood		−1065.310		−1104.011		−628.157	
Chi-Sq		85.290		50.848		788.694	
DF		4		5		6	
P value		0.000***		0.000***		0.000***	
Measures of association							
Somers’ D		0.30		0.19		0.74	
Goodman–Kruskal γ		0.30		0.20		0.75	
Kendall’s τ_*A*_		0.15		0.10		0.32	

Prediction pair type		Count	Proportion	Count	Proportion	Count	Proportion
Concordant pairs		418,855	0.646	383,544	0.576	500,456	0.862
Discordant pairs		223,945	0.345	255,698	0.384	70,286	0.121
Tied pairs		5386	0.008	26,410	0.040	9,500	0.016
Total		648,186	1.000	665,652	1.000	580,242	1.000

^a^Reference case (i.e., Success)

^b^Reference level

**Table 5:** Successive binary logistic regression analyses (not-for-profit institutions, *n*_2_ = 439)

		Model (1)		Model (2)		Model (3)	
Response variable	(Level)	B1	(0, 1^a^)	B2	(0, 1^a^)	B3	(0, 1^a^)
Level	(Count)	0	(205)	0	(374)	0	(331)
		1	(234)	1	(65)	1	(41)
		Total	(439)	Total	(439)	Total	(372^c^)
Predictor variable	(Level)	X1		X1		X1	
		T1	U^b^, R	T1	U^b^, R	T1	U^b^, R
		T2	Sg^b^, Cs, Cd	T2	Sg^b^, Cs, Cd	T2	Sg^b^, Cd
				B1	(0^b^, 1)	B1	(0^b^, 1)
						B2	(0^b^, 1)

Variable	Level	Coefficient (Z value)	Odds ratio	Coefficient (Z value)	Odds ratio	Coefficient (Z value)	Odds ratio
Intercept		−1.291 (−3.91)		−2.374 (−5.27)		−4.445 (−5.75)	
X1		0.337 (7.59***)	1.40	0.110 (3.00***)	1.12	0.092 (1.86*)	1.10
T1	R	−0.122 (−0.52)	0.88	0.650 (1.82*)	1.92	0.569 (1.07)	1.77
T2	Cs	0.766 (1.95*)	2.15	−0.904 (−1.29)	0.40		
	Cd	0.607 (1.89*)	1.83	0.269 (0.67)	1.31	0.865 (1.37)	2.38
B1	1			−0.769 (−2.39**)	0.46	−0.443 (−0.97)	0.64
B2	1					2.803 (7.19***)	16.50
Model significance test							
Log likelihood		−254.046		−171.189		−92.820	
Chi-Sq		98.574		25.799		72.502	
DF		4		5		5	
P value		0.000***		0.000***		0.000***	
Measures of association							
Somers’ D		0.55		0.39		0.73	
Goodman–Kruskal γ		0.55		0.39		0.73	
Kendall’s τ_*A*_		0.27		0.10		0.14	

Prediction pair type		Count	Proportion	Count	Proportion	Count	Proportion
Concordant pairs		36,985	0.771	16,712	0.687	11,650	0.858
Discordant pairs		10,759	0.224	7272	0.299	1800	0.133
Tied pairs		226	0.005	326	0.013	121	0.009
Total		47,970	1.000	24,310	1.000	13,571	1.000

^a^Reference case (i.e., Success)

^b^Reference level

^c^A partial sample composed of 67 observations accompanied with T2 = Cs is discarded because all these observations have B3 = 0 solely

#### Model estimation (1): input versus performance

As shown in Table 3, X1 is a statistically significant predictor variable in Model (1) with a positive (+) estimated coefficient $\hat{\beta }_{1}$ = 0.283 and Z value = 10.04***. Based on the odds ratio exp($\hat{\beta }_{1}$) = 1.33, we can interpret that patent registration performance creation success probability odds ratio increases 1.33-fold with 1 unit increment in X1 (Hosmer and Lemeshow 2000; Montgomery et al. 2001; Minitab 2005; IBM SPSS 2009).

Here, an interesting point is found that changes of the estimated coefficients of X1 are (+) 0.283*** → (+) 0.028 → (+) 0.021 along with the successive model extension from Model (1) to Model (3). As seen, all three estimated coefficients of X1 have positive signs (+) consistently, but both the absolute values and the Z values of these decrease monotonically according to the successive model extension. In addition, the statistical significance of the estimated coefficient of X1 is confirmed in Model (1) solely, and then X1 becomes statistically insignificant in the two other models, Model (2) and Model (3). Therefore, a phenomenon can be pinpointed that R&D inputs can exert their influence more on the chronologically adjacent short-term, technical output performance factor B1. Afterwards, their influence diminishes against the mid-term, economic outcome B2 and the long-term, socioeconomic impact B3.

#### Model estimation (2): external influences

Table 3 presents three estimated coefficients associated with the external influence variable T1 in Model (1), except for the reference level T1 = L. The level T1 = U has a positive (+) estimated coefficient $\hat{\beta }_{{{\text{T}}1^{\text{U}} }}$  = 0.343 and Z value = 1.65*, which is statistically significant to the response variable B1. Because the level of T1 = U yields a positive (+) estimated coefficient, the odds ratio exp($\hat{\beta }_{{{\text{T}}1^{\text{U}} }}$) = 1.41 is larger than one. Thus, patent registration performance creation success probability odds ratio increases 1.41-fold when the level of T1 changes from the reference level T1 = L to T1 = U. Therefore, patent registration performance creation success probability is sensitive to institution types. Specifically, the university institution type shows the best probability, and the three other institution types (i.e., research laboratory, large company and SMS) are not statistically significantly different.

On the other hand, all three estimated coefficients associated with T1 in Model (2) are statistically significant to the response variable B2: (1) the level of T1 = S has a positive (+) estimated coefficient $\hat{\beta }_{{{\text{T}}1^{\text{S}} }}$ = 0.702 and Z value = 5.39***, (2) the level of T1 = R has a negative (-) estimated coefficient $\hat{\beta }_{{{\text{T}}1^{\text{R}} }}$  = −0.927 and Z value = −4.77***, and (3) the level of T1 = U has a negative (-) estimated coefficient $\hat{\beta }_{{{\text{T}}1^{\text{U}} }}$ = −1.770 and Z value = −5.48***. It is noted that there is a monotonically decreasing sequence of exp($\hat{\beta }_{{{\text{T}}1^{\text{S}} }}$) = 2.02*** → exp($\hat{\beta }_{{{\text{T}}1^{\text{R}} }}$) = 0.40*** → exp($\hat{\beta }_{{{\text{T}}1^{\text{U}} }}$) = 0.17***. It implies that the sales performance creation success probability odds ratio decreases significantly when the institution type changes from for-profit institutions to not-for-profit institutions as follows: SME (1st) → Large Company (2nd) → Research Laboratory (3rd) → University (4th). It is verified that sales performance creation success probability is very sensitive to institution types. In particular, among a total of seven predictor variables in Model (2), the level of T1 = S has the largest odds ratio. It means that a certain GSP’s sales performance can be greatly enhanced when it is conducted by an SME.

In Table 3, Model (3) shows similar results to Model (2). For the response variable B3, the three consecutive odds ratios decrease monotonically, exp($\hat{\beta }_{{{\text{T}}1^{\text{S}} }}$) = 1.54** → exp($\hat{\beta }_{{{\text{T}}1^{\text{R}} }}$) = 0.74 → exp($\hat{\beta }_{{{\text{T}}1^{\text{U}} }}$) = 0.39*. Even though there is no statistically significant difference between research laboratory and large company type, new employment performance creation success probability odds ratios decrease along with the changes of institution types as follows: SME (1st) → Large Company (2nd) → Research Laboratory (3rd) → University (4th). As we can see, the ranks are identical to the B2 related odds ratios shown above. Consequently, an SME performs best in the aspect of sales and new employment. Meantime, university is excellent in the short-term, technical output factor (i.e., patent registration).

As for the external influence variable T2, two estimated coefficients are presented in Model (1) of Table 3, except for the reference level T2 = Sg. The level of T2 = Cd has a positive (+) estimated coefficient $\hat{\beta }_{{{\text{T2}}^{\text{Cd}} }}$  = 0.419 and Z value = 2.53**, which is statistically significant to the response variable B1. When we see the odds ratio, the patent registration performance creation success probability odds ratio increases 1.52-fold with the change from the reference level T2 = Sg to T2 = Cd (i.e., exp($\hat{\beta }_{{{\text{T2}}^{\text{Cd}} }}$) = 1.52). Thus, patent registration performance creation success probability is affected by R&D collaboration types, and R&D collaboration with the different type institutions can promote this probability.

Likewise, in Model (3), the level of T2 = Cd has a positive (+) estimated coefficient $\hat{\beta }_{{{\text{T2}}^{\text{Cd}} }}$ = 0.516 and Z value = 2.19**, which is statistically significant to the response variable B3. New employment performance creation success probability odds ratio increases 1.68-fold with the change from the reference level T2 = Sg to T2 = Cd (i.e., exp($\hat{\beta }_{{{\text{T2}}^{\text{Cd}} }}$) = 1.68). In summary, R&D collaboration with the different types of institutions promotes the performance of both patent registration and new employment.

#### Model estimation (3): performance chain

This section presents a comprehensive investigation on how closely the time-ordered previous and subsequent performance factors relate to one another. First, we try to identify the relationship between the response variable B2 and the predictor variable B1 in Model (2) of Table 3. The predictor variable B1 has a positive (+) estimated coefficient $\hat{\beta }_{\text{B1}}$ = 0.376 and Z value = 3.79***, which is statistically significant to the response variable B2. Sales performance creation success probability odds ratio increases 1.46-fold when B1 changes from the reference level B1 = 0 to B1 = 1 (i.e., exp($\hat{\beta }_{\text{B1}}$) = 1.46). Thus, a higher sales performance creation success probability is detected when an observation creates patent registration performance in advance.

Furthermore, referring to Model (3) in Table 3, it is verified that two predictor variables B1 and B2 are statistically significant to the response variable B3 simultaneously. The predictor variable B2 has a positive (+) estimated coefficient $\hat{\beta }_{{{\text{B}}2}}$ = 3.603, Z value = 21.65*** and exp($\hat{\beta }_{{{\text{B}}2}}$) = 36.72. Therefore, new employment performance creation success probability odds ratio increases as much as 36.72-fold when B2 changes from the reference level B2 = 0 to B2 = 1. Most notably, among the eight predictor variables in Model (3), B2 has the largest values of estimated coefficient, Z value and odds ratio. This finding implies that new employment performance creation success probability increases drastically by achieving sales performance beforehand. In the comparison with B2, B1 survives as a significant predictor variable to the response variable B3, even though both the absolute value and the statistical significance of the estimated coefficient are weakened slightly. Specifically, B1 has a positive (+) estimated coefficient $\hat{\beta }_{{{\text{B}}1}}$ = 0.420, Z value = 3.06***, and the odds ratio exp($\hat{\beta }_{{{\text{B}}1}}$) = 1.52. Therefore, the preceding performance creation of sales and patent registration can act as a catalyst for creating the subsequent performance of new employment. In summarizing the series of three successive binary logistic regression models analyzed from Model (1) to Model (3) in Table 3, the research model’s performance chain structure showing B3 ← B2 ← B1 ← X1 is clearly identified, accompanied with statistical significance.

### Logistic regression analysis: sample split (1) (for-profit institutions) (n_1_ = 1637)

#### Model structure

Table 4 shows the primary results from analyzing three successive binary logistic regression models from Model (1) to Model (3) using the partial sample of *n*_1_ = 1637 observations associated with for-profit institutions only. As aforementioned, the two samples analyzed in “Logistic regression analysis: the whole sample (n = 2076)” and “Logistic regression analysis: sample split (1) (for-profit institutions) (n_1_ = 1637)” sections are overlapped approximately 80 % (i.e., *n* and *n*_1_), as the results and interpretation in “Logistic regression analysis: sample split (1) (for-profit institutions) (n_1_ = 1637)” section are very similar to the explanation in “Logistic regression analysis: the whole sample (n = 2076)” section. Thus, only distinctive features of Table 4 are explained briefly below in the comparison with Table 3.

Regarding the two external influence variables, the levels of T1 = L and T2 = Sg are defined as the reference levels in Model (1) of Table 4. Since only for-profit-institutions observations are extracted, the external influence variable T1 becomes a 2-level categorical variable (i.e., T1 = L and T1 = S). The number of observations achieving performance (i.e., the number of observations with the binary response variable equal to one) are counted as follows: (1) Model (1), B1 = 1, 671 (671/1637 × 100 = 40.99 %), (2) Model (2), B2 = 1, 753 (46.00 %), and (3) Model (3), B3 = 1, 519 (31.70 %). With respect to for-profit institutions, the proportion of observations creating sales performance exceeds the proportion of observations creating patent registration performance (i.e., 46.00 > 40.99 %).

#### Model estimation (1): input versus performance

In Model (1) of Table 4, X1 is statistically significant with a positive (+) estimated coefficient $\hat{\beta }_{1}$ = 0.242 and Z value = 6.44***. Based on the odds ratio exp($\hat{\beta }_{1}$) = 1.27, we can interpret that patent registration performance creation success probability odds ratio increases 1.27-fold with 1 unit increment in X1. Similar to the results from the analyses using the whole sample of *n* = 2076, only Model (1) shows the statistical significance of X1 with the expected positive (+) sign. Again, in the case of for-profit institutions, the influence of R&D inputs is confined within the chronologically adjacent short-term, technical output performance factor B1.

#### Model estimation (2): external influences

Table 4 presents only one estimated coefficient associated with the external influence variable T1, except for the reference level T1 = L. In Model (2), the level T1 = S has a positive (+) estimated coefficient $\hat{\beta }_{{{\text{T}}1^{\text{S}} }}$ = 0.644, Z value = 4.76*** and the odds ratio for the response variable B2, exp($\hat{\beta }_{{{\text{T}}1^{\text{S}} }}$) = 1.90. Also, Model (3) shows similar results such as $\hat{\beta }_{{{\text{T}}1^{\text{S}} }}$  = 0.372, Z value = 1.87* and the odds ratio for the response variable B3, exp($\hat{\beta }_{{{\text{T}}1^{\text{S}} }}$) = 1.45. So, an SME performs better than a large company for creating sales and new employment performance.

In Table 4, two estimated coefficients are presented related to T2, except for the reference level T2 = Sg. In Model (1), the level of T2 = Cd has a positive (+) estimated coefficient $\hat{\beta }_{{{\text{T2}}^{\text{Cd}} }}$  = 0.359 and Z value = 1.84*, which is statistically significant to the response variable B1. Patent registration performance creation success probability odds ratio increases 1.43-fold with the change from the reference level T2 = Sg to T2 = Cd (i.e., exp($\hat{\beta }_{{{\text{T2}}^{\text{Cd}} }}$) = 1.43). Similarly, in Model (3), the level of T2 = Cd has a positive (+) estimated coefficient $\hat{\beta }_{{{\text{T2}}^{\text{Cd}} }}$ = 0.512 and Z value = 1.98**, which is statistically significant to the response variable B3. New employment performance creation success probability odds ratio increases 1.67-fold with the change from the reference level T2 = Sg to T2 = Cd (i.e., exp($\hat{\beta }_{{{\text{T2}}^{\text{Cd}} }}$) = 1.67). In the case of for-profit-institutions, R&D collaboration with the different types of institutions enhances the performance of both patent registration and new employment.

#### Model estimation (3): performance chain

In Model (2) of Table 4, the predictor variable B1 has a positive (+) estimated coefficient $\hat{\beta }_{\text{B1}}$ = 0.514 and Z value = 4.87***, which is statistically significant to the response variable B2. Sales performance creation success probability odds ratio increases 1.67-fold when B1 changes from the reference level B1 = 0 to B1 = 1 (i.e., exp($\hat{\beta }_{\text{B1}}$) = 1.67). In Model (3), two predictor variables B1 and B2 are statistically significant to the response variable B3 simultaneously. In particular, the predictor variable B2 has a positive (+) estimated coefficient $\hat{\beta }_{{{\text{B}}2}}$  = 3.733, Z value = 19.79*** and exp($\hat{\beta }_{{{\text{B}}2}}$) = 41.79. Therefore, new employment performance creation success probability odds ratio increases drastically as much as 41.79-fold, when B2 changes from the reference level B2 = 0 to B2 = 1. Also, among the six predictor variables in Model (3), B2 has the largest values of estimated coefficient, Z value and odds ratio. Even though the influence is diminished slightly compared with B2, B1 remains as a significant predictor variable to the response variable B3. The predictor variable B1 has a positive (+) estimated coefficient $\hat{\beta }_{{{\text{B}}1}}$ = 0.497 and Z value = 3.40***, and exp($\hat{\beta }_{{{\text{B}}1}}$) = 1.64. Hence, new employment performance creation success probability is heavily sensitive to the two predecessor performance success-failure within for-profit-institutions’ GSPs. As described in detail in “Model estimation (3): performance chain” section, the performance chain structure of B3 ← B2 ← B1 ← X1 is revealed once again.

### Logistic regression analysis: sample split (2) (not-for-profit institutions) (n_2_ = 439)

#### Model structure

Table 5 shows the results from analyzing three successive binary logistic regression models from Model (1) to Model (3) using the partial sample of *n*_2_ = 439 observations conducted by not-for-profit institutions only. As explained below, the R&D performance creation behavior of not-for-profit institutions is clearly distinctive from the for-profit institutions’ behavior identified in “Logistic regression analysis: the whole sample (n = 2076)” section. Meanwhile, it is noted that 67 observations are eliminated from the partial sample of *n*_2_ = 439, and the remaining 372 observations are used to analyze Model (3) in Table 5. Since the removed observations have T2 = Cs and the response variable B3 = 0 simultaneously, it is not possible to use them for estimating the coefficient of $\beta_{{{\text{T2}}^{\text{Cs}} }}$.

As for the two external influence variables T1 and T2, the levels of T1 = U and T2 = Sg are defined as the reference levels in Model (1) of Table 5. Because not-for-profit institutions’ observations are extracted solely, the external influence variable T1 becomes a 2-level categorical variable (i.e., T1 = U and T1 = R). The number of observations achieving performance are summarized as follows: (1) Model (1), B1 = 1, 234 (234/439 × 100 = 53.30 %), (2) Model (2), B2 = 1, 65 (14.81 %), and (3) Model (3), B3 = 1, 41 (41/372 × 100 = 11.02 %). Compared with the patent registration proportion of for-profit institutions, not-for-profit institutions show comparatively larger proportion (i.e., 53.30 > 40.99 %). On the contrary, the two other proportions are considerably smaller than the for-profit institutions’ corresponding proportions (i.e., sales 14.81 < 46.00 % and new employment 11.02 < 31.70 %). In the case of not-for-profit institutions, another feature is that patent registration proportion 53.30 % is relatively high, but the two subsequent proportions drop sharply (i.e., 53.30% → 14.81 % → 11.02 %).

#### Model estimation (1): input versus performance

In Model (1) of Table 5, X1 is statistically significant with a positive (+) estimated coefficient $\hat{\beta }_{1}$ = 0.337 and Z value = 7.59***. Based on the odds ratio exp($\hat{\beta }_{1}$) = 1.40, so we can interpret that patent registration performance creation success probability odds ratio increases 1.40-fold with 1 unit increment in X1. In the case of not-for-profit institutions, there is a distinctive relationship between the R&D input variable X1 and the three R&D performance binary variables B1, B2 and B3. When the models are extended successively as Model (1) → Model (2) → Model (3), the estimated coefficient $\hat{\beta }_{ 1}$ is calculated consistently accompanied with statistical significance and the expected positive (+) sign as follows: (+) 0.337*** → (+) 0.110*** → (+) 0.092*. This is clearly different from the for-profit institutions’ pattern explained in “Model estimation (1): input versus performance” section. However, both the absolute values and the Z values of these estimates decrease monotonically according to the successive model extension. Consequently, R&D inputs can exert their influence throughout the entire R&D performance chain path from the short-term, technical output factor B1 to the long-term, socioeconomic impact factor B3 via the mid-term, economic outcome factor B2. However, it is noted that R&D inputs’ influence reduces gradually when the performance factors move forward along with the chain path.

#### Model estimation (2): external influences

In Model (2) of Table 5, the level T1 = R has a positive (+) estimated coefficient $\hat{\beta }_{{{\text{T}}1^{\text{R}} }}$ = 0.650, Z value = 1.82* and exp($\hat{\beta }_{{{\text{T}}1^{\text{R}} }}$) = 1.92. Compared with university type, research laboratory shows a higher sales performance creation success probability. When the two estimated coefficients are examined related to T2, the statistical significance is found in Model (1) only. In Model (1), the level of T2 = Cs has a positive (+) estimated coefficient $\hat{\beta }_{{{\text{T2}}^{\text{Cs}} }}$ = 0.766 and Z value = 1.95*, and the level of T2 = Cd has a positive (+) estimated coefficient $\hat{\beta }_{{{\text{T2}}^{\text{Cd}} }}$ = 0.607 and Z value = 1.89*. Hence, patent registration performance creation success probability odds ratio increases: (1) 2.15-fold with the change from the reference level T2 = Sg to T2 = Cs (i.e., exp($\hat{\beta }_{{{\text{T2}}^{\text{Cs}} }}$) = 2.15), and (2) 1.83-fold with the change from the reference level T2 = Sg to T2 = Cd (i.e., exp($\hat{\beta }_{{{\text{T2}}^{\text{Cd}} }}$) = 1.83). In the case of not-for-profit institutions, patent registration performance can be improved by controlling R&D collaboration types. Specifically, the two R&D collaboration types denoted by Cs and Cd can lead to superior patent registration performance.

#### Model estimation (3): performance chain

When we examine the relationship between the response variable B3 and the two predictor variable B1 and B2 of Model (3) in Table 5, B2 is statistically significant to the response variable B3 only. The predictor variable B2 has a positive (+) estimated coefficient $\hat{\beta }_{{{\text{B}}2}}$ = 2.803, Z value = 7.19*** and exp($\hat{\beta }_{{{\text{B}}2}}$) = 16.50. So, new employment performance creation success probability odds ratio increases 16.50-fold when B2 changes from the reference level B2 = 0 to B2 = 1. Among the five predictor variables in Model (3), B2 has the largest values of estimated coefficient, Z value and odds ratio. Thus, new employment performance can be improved by generating sales performance in advance. However, the odds ratio exp($\hat{\beta }_{{{\text{B}}2}}$) = 16.50 of not-for-profit institutions is much less than the odds ratio exp($\hat{\beta }_{{{\text{B}}2}}$) = 41.79 of for-profit institutions. It indicates that the sensitivity of new employment performance creation success probability decreases in the case of not-for-profit institutions.

Unlike for-profit institutions, we cannot derive a statistically significant positive (+) estimated coefficient of the predictor variable B1 in the two models, Model (2) and Model (3) in Table 5. In contrast to our expectation, Model (2) shows the negative (-) estimated coefficient $\hat{\beta }_{{{\text{B}}1}}$ = −0.769 and Z value = −2.39**. Associated with not-for-profit institutions, two disconnected relationships are detected: B3 ← B2 ← X1 and B1 ← X1 performance chains. Therefore, it is interpreted that not-for-profit institutions do not greatly link patent registration to sales, as compared with for-profit institutions.

## Conclusions

Based on typical R&D logic models, various types of R&D performance factors can be evaluated more systematically within national technology innovation R&D programs, and some useful policy implications can be derived for restructuring subsequent R&D programs as well as R&D budget allocations more effectively. However, due to the nonnormal sample characteristics and the difficulty of dealing with the time lag between R&D inputs and performance, related literature is still limited especially associated with empirical analyses presenting both relevant research models and practical implications.

The present study analyzed a sample of *n* = 2076 completed GSPs within the representative national technology innovation R&D program. In particular, the present study verified differences in R&D performance creation behavior between for-profit institutions and not-for-profit institutions within the program. Methodologically, a series of successive binary logistic regression models was proposed, and the sample was split into two mutually exclusive subordinate datasets to compare R&D performance creation behavior between the two types of institution more accurately. Major results and implications of the present study are summarized as follows. First, the sustainability of the government R&D subsidy was relatively weaker for creating R&D performance within for-profit institutions. On the other hand, the government funds exerted its influence throughout the entire performance chain path of not-for-profit institutions. From the sustainability perspective, it might be desirable to invest more government R&D budget into GSPs managed by not-for-profit institutions. Also, practitioners should carefully select performance-oriented GSPs in building subsequent R&D programs considering this behavioral characteristic. Second, on the whole, because of the strong relationship between the two directly connected performance factors, we need to encourage a sequential performance creation as much as possible to extend a GSP R&D performance creation life cycle. Specifically, the for-profit institutions’ performance creation behavior conformed exactly to the stepwise chain structure. In contrast, not-for-profit institutions showed somewhat a discontinuous pattern. In particular, we discovered an undesirable pattern of not-for-profit institutions in which the technical output (i.e., patent registration) was not linked to the economic outcome (i.e., sales). Therefore, intensive efforts should be required to utilize patent registration to promote sales performance more frequently. Third, for-profit institutions achieved higher performance levels of patent registration and new employment simultaneously through R&D collaboration with the different types of institutions (i.e., T2 = Cd). However, not-for-profit institutions showed higher performance of patent registration only through R&D collaboration with the different types of institutions. Thus, for-profit institutions need to take advantage of the collaborative activities with not-for-profit institutions to enhance patent registration performance. This strategy can extend for-profit institutions’ performance creation life cycle gradually up to the economic and the socioeconomic performance levels. Meanwhile, both for-profit institutions and not-for-profit institutions should make efforts to generate sales performance through R&D collaboration. Fourth, SMEs performed best in the aspect of sales and new employment, and universities excelled in the performance of patent registration. Thus, each institution type has its own performance factors with competitive advantages. Therefore, it is necessary to select appropriate GSPs for satisfying the program-level objectives effectively.

The present study did not consider other intangible R&D inputs such as inherent R&D capability owned by the institutions and accumulated R&D knowledge and experience through R&D activities in the past. Therefore, these intangible R&D inputs should be reflected in a future modification of the research model. Furthermore, ordinal logistic and Poisson regression models can be incorporated into the successive regression analyses procedure to accommodate more finely categorized values of the performance variables.

## Appendix

As mentioned before, associated with the design of successive binary logistic regression models, all the mathematical details are arranged into the appendix for the readership not to wade through all the detailed equations. First of all, the two external influence variables T1 and T2 are defined as Eqs. (1) and (2) respectively.

1 $${\text{T}}1_{i} = \left\{ {\begin{array}{l} {{\text{L}}\quad {\text{if}}\;{\text{the }}i{\text{th observation by `Large Company',}}} \hfill \\ {{\text{U}}\quad {\text{if}}\;{\text{the }}i{\text{th observation by `University',}}} \hfill \\ {\text{R}}\quad {\text{if}}\;{\text{the }}i{\text{th observation by `Research Laboratory',}} \hfill \\ {\text{S}}\quad {\text{otherwise}}\;({\text{i}} . {\text{e}} . ,\;`{\text{SME')}} \hfill \\ \end{array} } \right.$$

2 $${\text{T2}}_{i} = \left\{ {\begin{array}{l} {{\text{Sg}}\quad {\text{if}}\;{\text{the }}i{\text{th observation by `Single Institution R}}\& {\text{D',}}} \hfill \\ {{\text{Cs}}\quad {\text{if}}\;{\text{the }}i{\text{th observation by `R}}\& {\text{D Collaboration with the Same Type Institution',}}} \hfill \\ {{\text{Cd}}\quad {\text{otherwise}}\;({\text{i}} . {\text{e}} . , {\text{ `R}}\& {\text{D Collaboration with the Different Type Institution')}}} \hfill \\ \end{array} } \right.$$

Model (1) is a binary logistic regression model defined as Eq. (3), which explains the binary response variable B1 with the three predictor variables X1, T1 and T2. Therefore, Model (1) analyzes patent registration performance creation success-failure probability response to changes in the R&D budget, types of institution and types of R&D collaboration. As verified in “Empirical analysis” section, statistically significant positive (+) correlations existed among the three input variables X1, X2 and X3. Assuming that the multicollinearity problem could be caused by including the three input variables together in the regression model, Model (1) is established as a reduced model containing only one representative input variable X1. For interpretation purposes, Eq. (4) is derived from the logit transformation formula in which the logistic distribution link function is applied to the probability of B1_*i*_ = 1 (i.e., patent registration performance creation success probability π1_*i*_). Therefore, Eq. (4) becomes a general linear regression model, and its response variable is the natural log-transformation of the odds ratio of π1_*i*_.

3 $$\begin{aligned} \bullet \quad{\text{Model}}(1){:} & {\text{E(}}\left. {{\text{B}}1} \right|{\text{X}}1,\;{\text{T}}1,\;{\text{T2}}) \\ & =\, f({\text{X}}1,\;{\text{T}}1,\;{\text{T2}}) \\ & = \frac{{\exp (\beta_{0} + \beta_{1} {\text{X}}1 + \beta_{{{\text{T}}1}} {\text{T}}1 + \beta_{{{\text{T}}2}} {\text{T2}})}}{{1 + \exp (\beta_{0} + \beta_{1} {\text{X}}1 + \beta_{{{\text{T}}1}} {\text{T}}1 + \beta_{{{\text{T}}2}} {\text{T2}})}} \\ \end{aligned}$$

4 $${ \ln }_{\text{e}} \left( {\frac{{{{\uppi }}1_{i} }}{{1 - {{\uppi }}1_{i} }}} \right) = \beta_{0} + \beta_{1} {\text{X}}1 + \beta_{{{\text{T}}1}} {\text{T}}1 + \beta_{{{\text{T}}2}} {\text{T2}}$$

Since T1 is a 4-level categorical variable, Model (1) in Eq. (3) is converted to Eq. (5) based on the reference case and reference levels specified in “Empirical analysis” section. In Eq. (5), T1 is replaced by three binary variables ${\text{T1}}^{\text{U}}$, ${\text{T1}}^{\text{R}}$ and ${\text{T1}}^{\text{S}}$ where ${\text{T1}}^{\text{U}}$, ${\text{T1}}^{\text{R}}$ and ${\text{T1}}^{\text{S}}$ correspond to the three institution types, university, research laboratory and SME respectively. In the same context, Eq. (4) is converted to Eq. (6). Likewise, in Eqs. (5) and (6), T2 is replaced by two binary variables ${\text{T2}}^{\text{Cs}}$ and ${\text{T2}}^{\text{Cd}}$ where ${\text{T2}}^{\text{Cs}}$ and ${\text{T2}}^{\text{Cd}}$ correspond to the two R&D collaboration types, R&D collaboration with the same type institution and R&D collaboration with the different type institution respectively. The variable conversion related to the two categorical variables T1 and T2 is commonly applied from Model (2) to Model (3) as explained afterward.

5 $$\begin{aligned} \bullet {\text{Model}}(1){:} \quad& {\text{E}}\left( {\left. {{\text{B}}1} \right|{\text{X}}1,{\text{T1}}^{\text{U}} ,{\text{T1}}^{\text{R}} ,{\text{T1}}^{\text{S}} , {\text{ T2}}^{\text{Cs}} ,{\text{T2}}^{\text{Cd}} } \right) \\ & = f\left( {{\text{X}}1,{\text{T1}}^{\text{U}} ,{\text{T1}}^{\text{R}} ,{\text{T1}}^{\text{S}} , {\text{ T2}}^{\text{Cs}} ,{\text{T2}}^{\text{Cd}} } \right) \\ & = \frac{{\exp \left( {\beta_{0} + \beta_{1} {\text{X}}1 + \beta_{{{\text{T}}1^{\text{U}} }} {\text{T1}}^{\text{U}} + \beta_{{{\text{T}}1^{\text{R}} }} {\text{T1}}^{\text{R}} + \beta_{{{\text{T}}1^{\text{S}} }} {\text{T1}}^{\text{S}} + \beta_{{{\text{T}}2^{\text{Cs}} }} {\text{T}}2^{\text{Cs}} + \beta_{{{\text{T}}2^{\text{Cd}} }} {\text{T}}2^{\text{Cd}} } \right)}}{{1 + \exp \left( {\beta_{0} + \beta_{1} {\text{X}}1 + \beta_{{{\text{T}}1^{\text{U}} }} {\text{T1}}^{\text{U}} + \beta_{{{\text{T}}1^{\text{R}} }} {\text{T1}}^{\text{R}} + \beta_{{{\text{T}}1^{\text{S}} }} {\text{T1}}^{\text{S}} + \beta_{{{\text{T}}2^{\text{Cs}} }} {\text{T}}2^{\text{Cs}} + \beta_{{{\text{T}}2^{\text{Cd}} }} {\text{T}}2^{\text{Cd}} } \right)}} \\ \end{aligned}$$

6 $${ \ln }_{\text{e}} \left( {\frac{{{{\uppi }}1_{i} }}{{1 - {{\uppi }}1_{i} }}} \right) = \beta_{0} + \beta_{1} {\text{X}}1 + \beta_{{{\text{T}}1^{\text{U}} }} {\text{T1}}^{\text{U}} + \beta_{{{\text{T}}1^{\text{R}} }} {\text{T1}}^{\text{R}} + \beta_{{{\text{T}}1^{\text{S}} }} {\text{T1}}^{\text{S}} + \beta_{{{\text{T}}2^{\text{Cs}} }} {\text{T}}2^{\text{Cs}} + \beta_{{{\text{T}}2^{\text{Cd}} }} {\text{T}}2^{\text{Cd}}$$

According to the R&D performance creation chain structure depicted in Fig. 1, a series of two additional successive binary logistic regression models are developed from Model (2) in Eq. (7) to Model (3) in Eq. (8). As seen, Model (2) in Eq. (7) analyzes the relationship between the response variable B2 (i.e., sales performance creation success-failure binary variable) and another predictor variable B1. Eventually, Model (3) in Eq. (8) is regarded as a fully extended model that relates new employment performance creation success-failure binary variable B3, located at the ending point of the research model, to the remaining two binary performance predictor variables positioned ahead (i.e., B1 and B2).

7 $$\begin{aligned} \bullet {\text{Model}}(2){:} \quad& {\text{E}}\left( {{\text{B2}}|{\text{X}}1,{\text{T}}1,{\text{T2, B1}}} \right) \\ & = f({\text{X}}1,{\text{T}}1,{\text{T2, B1}}) \\ & = \frac{{\exp \left( {\beta_{0} + \beta_{1} {\text{X}}1 + \beta_{{{\text{T}}1}} {\text{T}}1 + \beta_{{{\text{T}}2}} {\text{T2}} + \beta_{\text{B1}} {\text{B1}}} \right)}}{{1 + \exp \left( {\beta_{0} + \beta_{1} {\text{X}}1 + \beta_{{{\text{T}}1}} {\text{T}}1 + \beta_{{{\text{T}}2}} {\text{T2}} + \beta_{\text{B1}} {\text{B1}}} \right)}} \\ \end{aligned}$$

8 $$\begin{aligned} \bullet {\text{Model}}(3){:} \quad & {\text{E}}\left( {\left. {\text{B3}} \right|{\text{X}}1,\;{\text{T}}1,\;{\text{T2,}}\;{\text{B1,}}\;{\text{B2}}} \right) \\ & = f\left( {{\text{X}}1,\;{\text{T}}1,\;{\text{T2,}}\;{\text{B1,}}\;{\text{B2}}} \right) \\ & = \frac{{\exp \left( {\beta_{0} + \beta_{1} {\text{X}}1 + \beta_{{{\text{T}}1}} {\text{T}}1 + \beta_{{{\text{T}}2}} {\text{T2}} + \beta_{\text{B1}} {\text{B1}} + \beta_{\text{B2}} {\text{B2}}} \right)}}{{1 + \exp \left( {\beta_{0} + \beta_{1} {\text{X}}1 + \beta_{{{\text{T}}1}} {\text{T}}1 + \beta_{{{\text{T}}2}} {\text{T2}} + \beta_{\text{B1}} {\text{B1}} + \beta_{\text{B2}} {\text{B2}}} \right)}} \\ \end{aligned}$$
